# Identification of a fatty acid metabolism-related gene signature to predict prognosis in stomach adenocarcinoma

**DOI:** 10.18632/aging.205823

**Published:** 2024-05-13

**Authors:** Lei Liu, Jing Sun, Changqing Zhong, Ang Zhang, Guodong Wang, Sheng Chen, Shuai Zhang, Min Wang, Lianyong Li

**Affiliations:** 1 Department of Gastroenterology, Strategic Support Force Medical Center, Beijing 100101, China; 2 Department of Spinal Surgery, Strategic Support Force Medical Center, Beijing 100101, China; 3 Department of Hematopathology, Strategic Support Force Medical Center, Beijing 100101, China

**Keywords:** fatty acid metabolism, STAD, riskscore model, nomogram, immunotherapy

## Abstract

Background: Fatty acid metabolism (FAM) contributes to tumorigenesis and tumor development, but the role of FAM in the progression of stomach adenocarcinoma (STAD) has not been comprehensively clarified.

Methods: The expression data and clinical follow-up information were obtained from The Cancer Genome Atlas (TCGA). FAM pathway was analyzed by gene set enrichment analysis (GSEA) and single-sample GSEA (ssGSEA) methods. Univariate Cox regression analysis was conducted to select prognosis genes. Molecular subtypes were classified by consensus clustering analysis. Furthermore, least absolute shrinkage and selection operator (Lasso) analysis was employed to develop a risk model. ESTIMATE and tumour immune dysfunction and exclusion (TIDE) algorithm were used to assess immunity. pRRophetic package was conducted to predict drug sensitivity.

Results: Based on 14 FAM related prognosis genes (FAMRG), 2 clusters were determined. Patients in C2 showed a worse overall survival (OS). Furthermore, a 7-FAMRG risk model was established as an independent predictor for STAD, with a higher riskscore indicating an unfavorable OS. High riskscore patients had higher TIDE score and these patients were more sensitive to anticancer drugs such as Bortezomib, Dasatinib and Pazopanib. A nomogram based on riskscore was an effective prediction tool applicable to clinical settings. The results from pan-cancer analysis supported a prominent application value of riskscore model in other cancer types.

Conclusion: The FAMRGs model established in this study could help predict STAD prognosis and offer new directions for future studies on dysfunctional FAM-induced damage and anti-tumor drugs in STAD disease.

## INTRODUCTION

Stomach adenocarcinoma (STAD) is a prevalent pathological tissue in stomach cancer, accounting for up to 95%, and the mortality of which ranks fourth in Global Cancer Statistics 2020 [1]. For early STAD, surgical resection is the treatment of choice, including total or partial gastrectomy. Lymph node dissection is often performed in conjunction with surgery to assess tumor spread [2]. Chemotherapy is usually used as adjuvant therapy in advanced stages or after surgery. First-line treatment options may include fluorouracil-based drugs (5-FU or tegafur) and platinum-based drugs (cisplatin or oxaliplatin). Radiotherapy is often used in combination with chemotherapy, especially in adjuvant therapy after surgery or for locally advanced tumors that cannot be surgically removed [3]. For HER2-positive STAD, targeted agents such as trastuzumab (Herceptin) may be used. In recent years, immune checkpoint inhibitors such as pembrolizumab (Opdivo) and pembrolizumab (Keytruda) have been used in certain types of advanced gastric adenocarcinoma [4]. There are several critical challenges in the current treatment of STAD. The first is the late diagnosis; many STAD patients are diagnosed at an advanced stage, by which time the tumor has usually metastasized and is more difficult to treat. Actually, it is the high recurrence rate, even after treatment, STAD still has a high recurrence rate, especially after surgical resection. The third is drug resistance and limited options for targeted therapy and immunotherapy [5, 6]. Currently, few gratifying results is found in predicting the prognosis of STAD sufferers with 5-year survival [7], although. To this end, developing potential factors to screen out the patient for individualized treatment is critical for the optimization of OS in STAD.

Mounting evidence implies that metabolic reprogramming plays a major part in the development of cancer for providing energy to biological behavior of tumors [8]. Among all metabolic progress, lipid metabolism, especially FAM, is vital for the reproduction of cancer cells [9]. Zaytseva et al. [10] reported that increased expression of fatty acid synthase in colorectal cancer is closely relevant to tumor progression and poor prognosis. Evidence also has shown that an abnormal activation of fatty acid oxidation is uncovered in various tumors, which are relevant to growth, invasiveness and radiochemotherapeutic resistance of cancer cells [11–13]. Given that FAM disorders take a bigger portion in cancer development, recent research has been more focused on the role of biological signaling pathway in regulating fatty acid metabolism. For example, Qu et al. [14] revealed that suppressed AMPK-GATA3-ECHS1 pathway could induce lipid accumulation and finally facilitate cell proliferation in clear cell renal cell carcinoma. Other studies also found that fatty acid synthesis as well as fatty acid uptake are motivated by mTOR signaling in cancers [15]. Luo et al.’s review also presented novel ideas that overcoming FA metabolic barriers is helpful for improving the effects of current immunotherapies [16]. FAM is also closely related to STAD progression, especially regulating STAD metastasis. It was found that the long noncoding RNA NEAT1 acts on the RPRD1B-regulated c-Jun/c-Fos/SREBP1 axis to promote fatty acid metabolism and lymph node metastasis in gastric cancer, a phenomenon that leads to primary tumor implantation into lymph nodes [17]. LINC00924-induced reprogramming of fatty acid metabolism through hnRNPC-regulated Mnk2 selective splicing promotes peritoneal metastasis of gastric cancer [18]. The FAM process is also a factor that influences immune cell function in STAD. Tissue-resident memory T (Trm) cells are key cells that influence the efficacy of immune checkpoint inhibitors in gastric cancer treatment. Fatty acid deficiency leads to Trm cell death [19]. Thus, targeting FAM pathways has been proven to be a prospective anticancer method.

Risk assessment models in bioinformatics (biosignatures) play a critical role in cancer research, especially in individualized treatment, prognosis prediction, and disease surveillance. These models analyze and integrate large amounts of data from patients - including gene expression, mutations, epigenetic changes, protein expression levels, and other clinical information-to predict patient response to specific treatments, disease progression, and the patient’s survival probability [20–22]. The Cox-nnet algorithm, an artificial neural network-based survival analysis method for prognostic prediction of high-throughput histological data, predicts survival trends in cancer patients from gene expression data [23]. The DeepSurv model, a Cox proportional risk model based on deep learning, is able to process individualized survival data to recommend the best treatment plan for patients [24]. These tools, unlike traditional tools, use high-throughput data as a fulcrum to reveal disease progression trends or treatment response in cancer patients from a genomic or proteomic perspective. Constructing a riskscore model has been developed into a practical means to assess patients’ prognosis, and is a promising means of precision-treatment. For example, a prognostic riskscore model with FAMRGs for predicting prognosis in colorectal cancer [25], lung adenocarcinoma [26] or glioma patients [27] has helped select different treatment drugs and regimens for high or low risk core grade patients. Nevertheless, there is still a paucity of research on the prognostic riskscore model of FAMRGs in STAD patients.

Here, we explored a FAM disorder-related molecular subtypes to predict OS of STAD patients. A prognostic signature (FAMRGs) model was established via univariate Cox regression analysis and Lasso regression analysis. We detected a potential for immunotherapy of the riskscore model based on tumor mutational burden (TMB), immune cell infiltration and TIDE score. Collectively, we established a riskscore signature for STAD patients, which may be treated as a potential therapeutic target for STAD.

## MATERIALS AND METHODS

### Study source

The expression data and clinical follow-up information were obtained from The Cancer Genome Atlas (TCGA, https://gdc.cancer.gov/, TCGA-STAD) database, containing gene expression profiles, copy number variation (CNV) data, single nucleotide mutation (SNV) data. Besides, STAD Gene Expression Omnibus (GEO, Gene Expression Omnibus) databases (GSE84437) were also enrolled. The following analysis were performed on Sangerbox (http://sangerbox.com/) [28] and R program.

### Data preprocessing

The following steps were done to the RNA-Seq data of TCGA-STAD:

(1) the samples with no clinical follow-up information were removed; (2) the samples with no survival time were removed; (3) the samples with no Status were removed; (4) the Ensembl was converted to Gene Symbol; and (5) the expression cases with multiple Gene Symbols were taken as their median.

The dataset of GSE84437 was processed in the following steps:

(1) the samples without clinical follow-up information were removed; (2) the samples without survival time and survival status were removed; (3) the probes were converted to Gene Symbol; (4) a probe corresponding to more than one gene was removed; and (5) the expression cases with more than one Gene Symbol were taken as their median.

### Gene set enrichment analysis (GSEA)

Batch effects or other unneeded variation in high-throughput data were eliminated applying “sva” package [29]. Then, the enrichment of FAM pathway in adjacent and tumor tissues was calculated in TCGA-STAD and GSE84437 datasets via GSEA [30].

### The single sample gene set enrichment analysis (ssGSEA)

In this paper, the ssGSEA was carried out by GSVA of R package [31] for transcriptome of STAD samples to evaluate FAM pathway score as well as pathway score difference between tumors and adjacent tumors. Normalized enrichment score (NES) >0 suggests pathway activation, while NES <0 suggests pathway inhibition.

### Cluster analysis

Based on FAM-related genes, grouping molecular subtypes was executed for TCGA-STAD and GSE84437 datasets via Consensus Cluster Plus package [32]. During analysis, TCGA samples were clustered through consistency clustering (ConsensusClusterPlus, specific parameters: clusterAlg = “pam”, distance = “spearman”, sampling was repeated 500 times, each sampling ratio was 0.8), and based on the cumulative distribution function (CDF) and CDF Delta area curve determine the optimal number of clusters. The Kaplan-Meier (K-M) survival curves were plotted to observe OS difference. Clustering effect was determined by principal component analysis (PCA).

### Analysis of cancer immune index

Estimation of Stromal and Immune cells in MAlignant Tumour tissues using Expression data (ESTIMATE) [33] was used to calculate the scale or abundance of major non-tumor components in a tumor sample, obtaining a general immune and stromal cell score. Microenvironment Cell Populations-counter (MCP-counter) [34] quantified the absolute abundance of eight immune cells and two stromal cells. Tumor immune dysfunction and exclusion (TIDE) (http://tide.dfci.harvard.edu/) website [35] was employed for assessing the response rate of immune checkpoint inhibitors (ICIs), while the relationship between TIDE index and riskscore was unveiled via Pearson correlation analysis.

### Establishment and assessment of a prognostic riskscore model for STAD

Construction of a riskscore signature was carried out based on Cox regression and Lasso method. At the same time, LASSO Cox regression analysis was executed to minimize the risk of overfitting. The following formula was applied to compute the riskscore for each patient.


riskscore=∑ βi×Expi

Here, β was the Cox regression coefficient of the corresponding genes, Exp i was the expression level of genes relevant to prognosis.

According to the survey package’s survey cut-point function, the optimal cutoff is discovered and the patients are assigned into high and low riskscore groups. K-M curves were plotted to estimate the OS in two groups. The timeROC package was used to determine the area under curve (AUC) to predict survival rate at 1 year, 3 years and 5 years, respectively.

### Independent prognostic and nomogram analysis

Cox regression analyses utilizing univariate and multivariate method were carried out to estimate if the riskscore can be acted as an independent factor for STAD prognosis. Over and above, a nomogram [36] is designed by consolidating riskscore level with common clinical indicators, and define a score for each indicator. Then, a total score can be obtained for every patient. Therefore, the probable survival time of the patient can be evaluated. The predictive efficiency of the nomogram was set to 1-year, 3- year, and 4-year OS. Decision curve analysis (DCA) analysis was employed to evaluate the prognostic value of the nomogram in clinical practice.

### Drug sensitivity

The pRRophetic R package [37] was used to predict two types of riskscore patient’s sensitivity to 62 traditional chemotherapy drugs, and the sensitivity to drug therapy was expressed by half maximal inhibitory concentration (IC50) values and uncovered by a graphical heatmap.

### Statistical analysis

The R program (version 4.1.2) as well as Sangerbox 3.0 (http://vip.sangerbox.com/) was used for statistical analysis. Statistical significance was defined as *p* < 0.05.

### Data availability statement

The datasets generated and/or analyzed during the current study are available in the (GSE84437) repository, (https://www.ncbi.nlm.nih.gov/geo/query/acc.cgi?acc=GSE84437), (GSE13522) repository, (https://www.ncbi.nlm.nih.gov/geo/query/acc.cgi?acc=GSE13522), (GSE78220) repository, (https://www.ncbi.nlm.nih.gov/geo/query/acc.cgi?acc=GSE78220) and (GSE91061) repository, (https://www.ncbi.nlm.nih.gov/geo/query/acc.cgi?acc=GSE91061).

## RESULTS

### Abnormally dysregulated fatty acid metabolism in STAD

**Figure 1:**
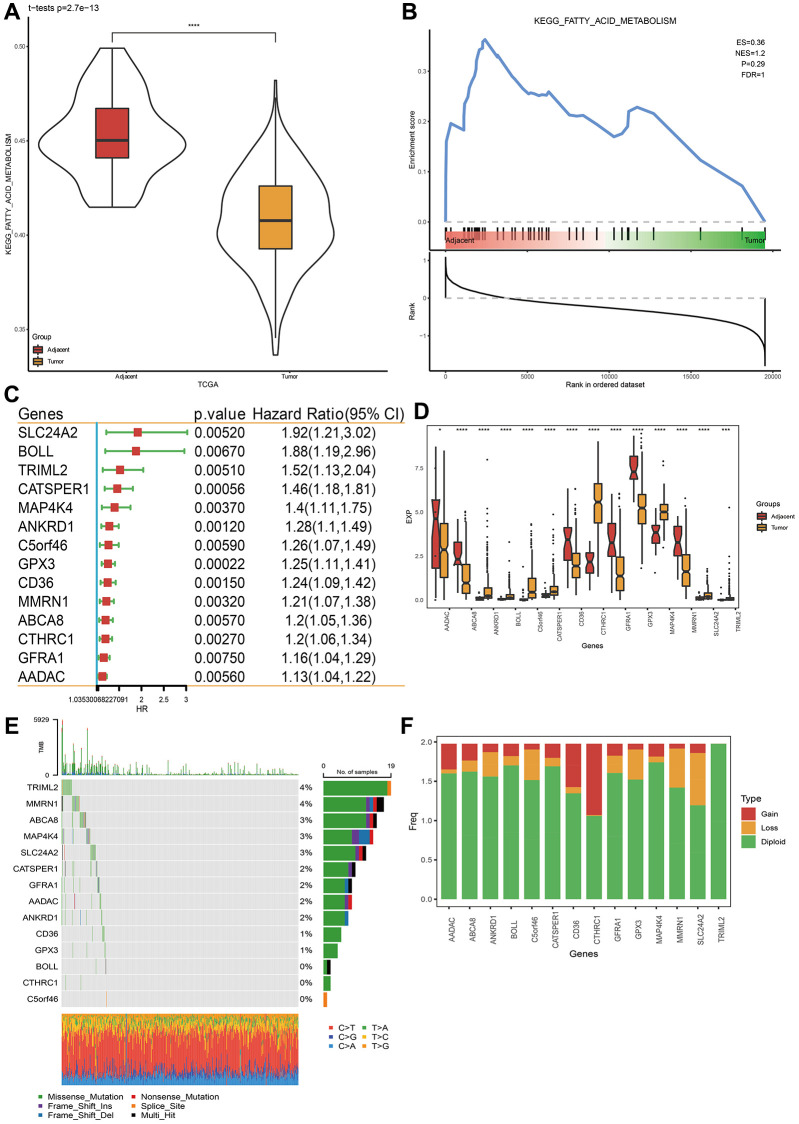
**Fatty acid metabolism pathway and related prognostic gene analysis.** (**A**, **B**) Fatty acid metabolism pathway analysis via ssGSEA and GSEA. (**C**) Genes closely related to FAM related gene pathway score in TCGA-STAD dataset. (**D**) Gene expression levels between STAD cancer tissue and para-carcinoma tissue. (**E**) Single nucleotide variation analysis of genes in TCGA-STAD dataset. (**F**) Copy number variation analysis. ^*^p < 0.05, ^***^p < 0.001, ^****^p < 0.0001.

Abundant researches have mentioned that FAM is essential for cancer progression and is constantly disturbed in various cancers [38]. Therefore, in this research, we firstly studied the status of FAM pathway in TCGA-STAD cohorts. Through the GSEA and ssGSEA method, FAM pathway is disordered in tumor tissues, compared with para-cancer samples (Figure 1A, 1B).

### Identification of FAM relevant genes closely related to STAD prognosis

In TCGA-STAD cohorts, we researched coding genes significantly correlated with FAM pathway scores, based on Spearman correlation analysis (|r| >0.35, *p* < 0.05) and univariate Cox analysis. Ultimately, 14 genes were predominantly correlated with prognosis, all of which were identified as risk factors as hazard ratios (HR) >1 (Figure 1C). Their expression levels showed remarkable differences between cancer and para-STAD samples (Figure 1D). Some of these genes were found gene mutation (Figure 1E). Furthermore, the CNV of these 14 genes revealed higher loss than gain (Figure 1F). These data demonstrated that dysregulated fatty acid metabolism was associated with STAD.

### Identification of molecular subtypes and biological role analysis

**Figure 2:**
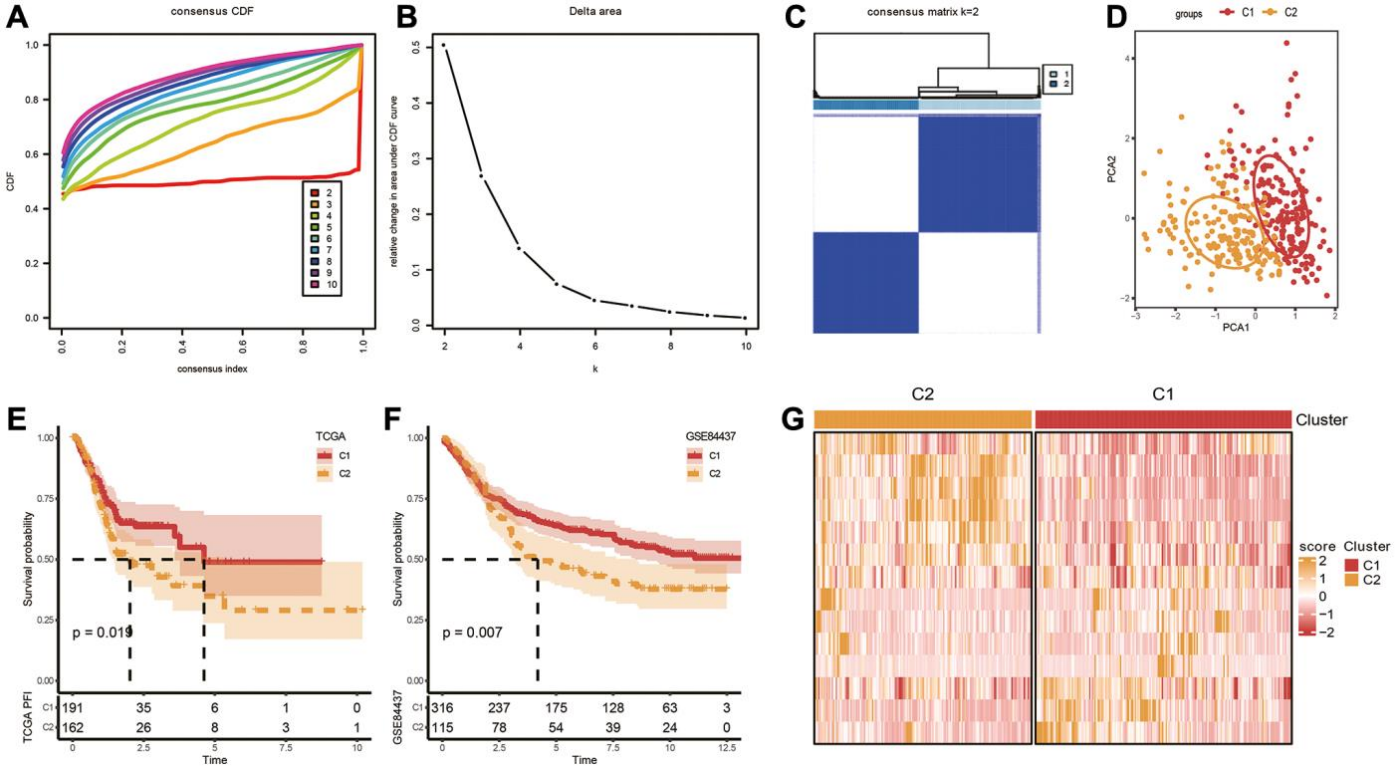
**Identification of molecular subtypes.** (**A**) Cumulative distribution function. (**B**) Delta area. (**C**, **D**) Heatmap and PCA plots of sample clustering when k = 2 in TCGA-STAD. (**E**, **F**) K-M survival analysis of C1 and C2 in TCGA-STAD and GSE84437 datasets. (**G**) Expression levels of 14 genes in C1 and C2 subtypes based on TCGA-STAD dataset.

Based on the aforementioned 14 genes, TCGA-STAD cohort were clustered, using the “Consensus Cluster Plus” package with K = 2 (Figure 2A, 2B). The cohort was separated into two molecular subtypes, called C1 and C2 (Figure 2C). To confirm the rationality of our classification, we performed PCA analysis using above 14 differentially expressed genes, and the results disclosed clear boundaries between the two subtypes (Figure 2D) Same classification and validation results were also got on the GSE84437 dataset (Supplementary Figure 1A). Next, the OS analysis found that subtype C1 exhibited a better prognosis than subtype C2 (Figure 2E, 2F). Additionally, the expression heatmap distribution of these 14 genes in TCGA data is shown in Figure 2G.

### Biological role analysis of molecular subtypes

**Figure 3:**
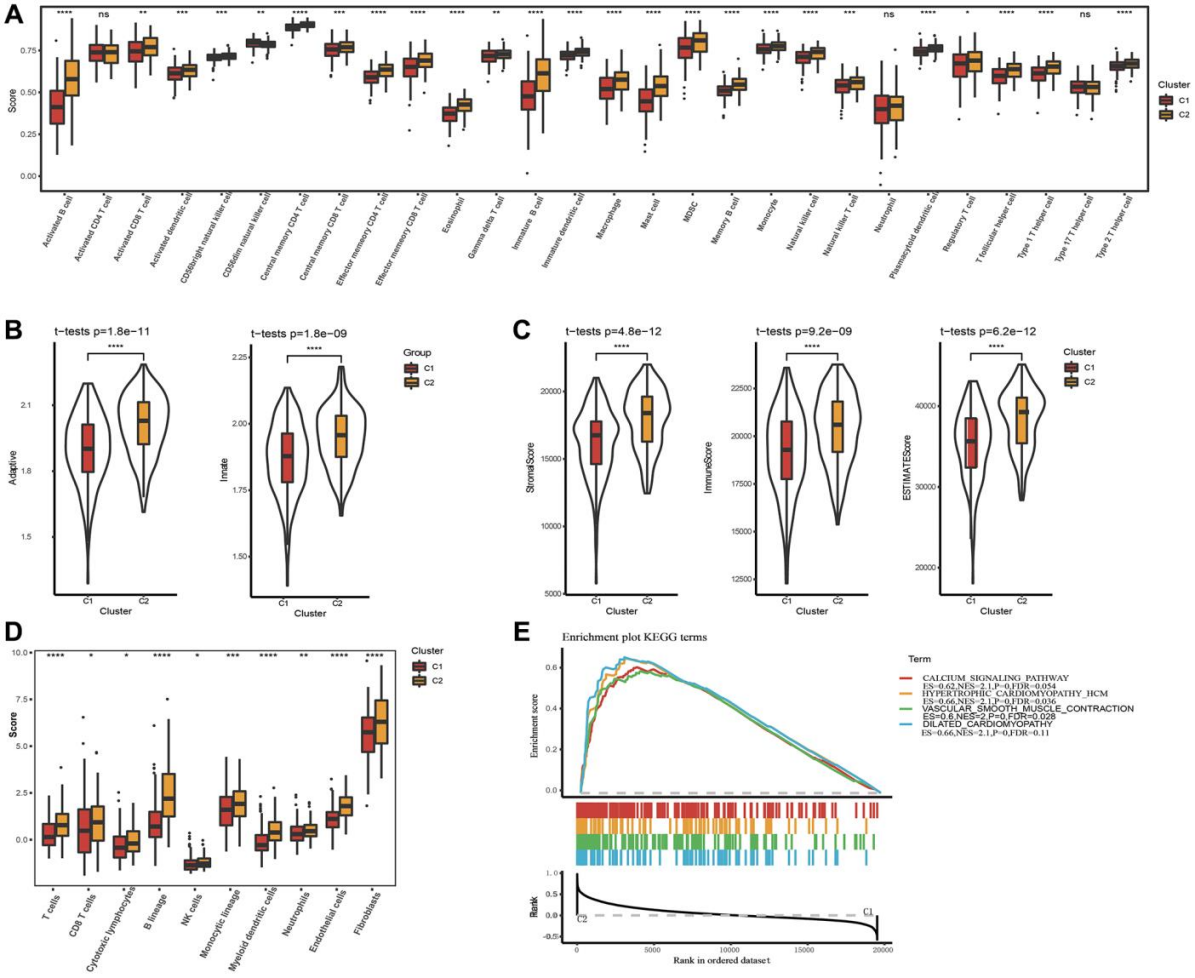
**Analysis of immune infiltration and gene set enrichment analysis.** (**A**) Analysis of 28 immune cells using CIBERSORT. (**B**) The distribution of innate and acquired immunity in TCGA-STAD dataset. (**C**) Analysis of immune infiltration using ESTIMATE. (**D**) Analysis of immune infiltration using MCP-counter. (**E**) GSEA pathways score analysis between C1 and C2 in TCGA-STAD. ^*^p < 0.05, ^**^p < 0.01, ^***^p < 0.001, ^***^p < 0.0001. Abbreviation: ns: no significance.

Immune infiltration in different subtypes was probed utilizing the ssGSEA method based on immune cell genes from literature [39], and compared the immune differences between two subtypes. The results showed that in the TCGA dataset, 24 of 28 immune cells had remarkable differences between two subtypes. The immune score of the C1 subtype with better prognosis was lower, both for innate immunity and acquired immunity (Figure 3A, 3B). We also used ESTIMATE and MCP-counter to perform immune scoring, and found that the immune score of the C1 subtype was still lower (Figure 3C, 3D), indicating that C1 had lower immune infiltration. Further GSEA analysis between molecular subtypes revealed that C2 subtype with poor prognosis is closely related to metabolic pathways including the HYPERTROPHIC_CARDIOMYOPATHY_HCM pathway and DILATED_CARDIOMYOPATHY pathway (Figure 3E). Similar GSEA analysis result was also seen on the GSE84437 dataset (Supplementary Figure 1B). Specifically, the lower degree of immune infiltration of the C1 subtype may affect its responsiveness to immunotherapy, and the metabolic pathways of the C2 subtype hint at the role of metabolic abnormalities in its tumor biology, which may be worse for this subtype related to the prognosis.

### Establishment of a clinical prognosis model applying FAMRGs and validation

**Figure 4:**
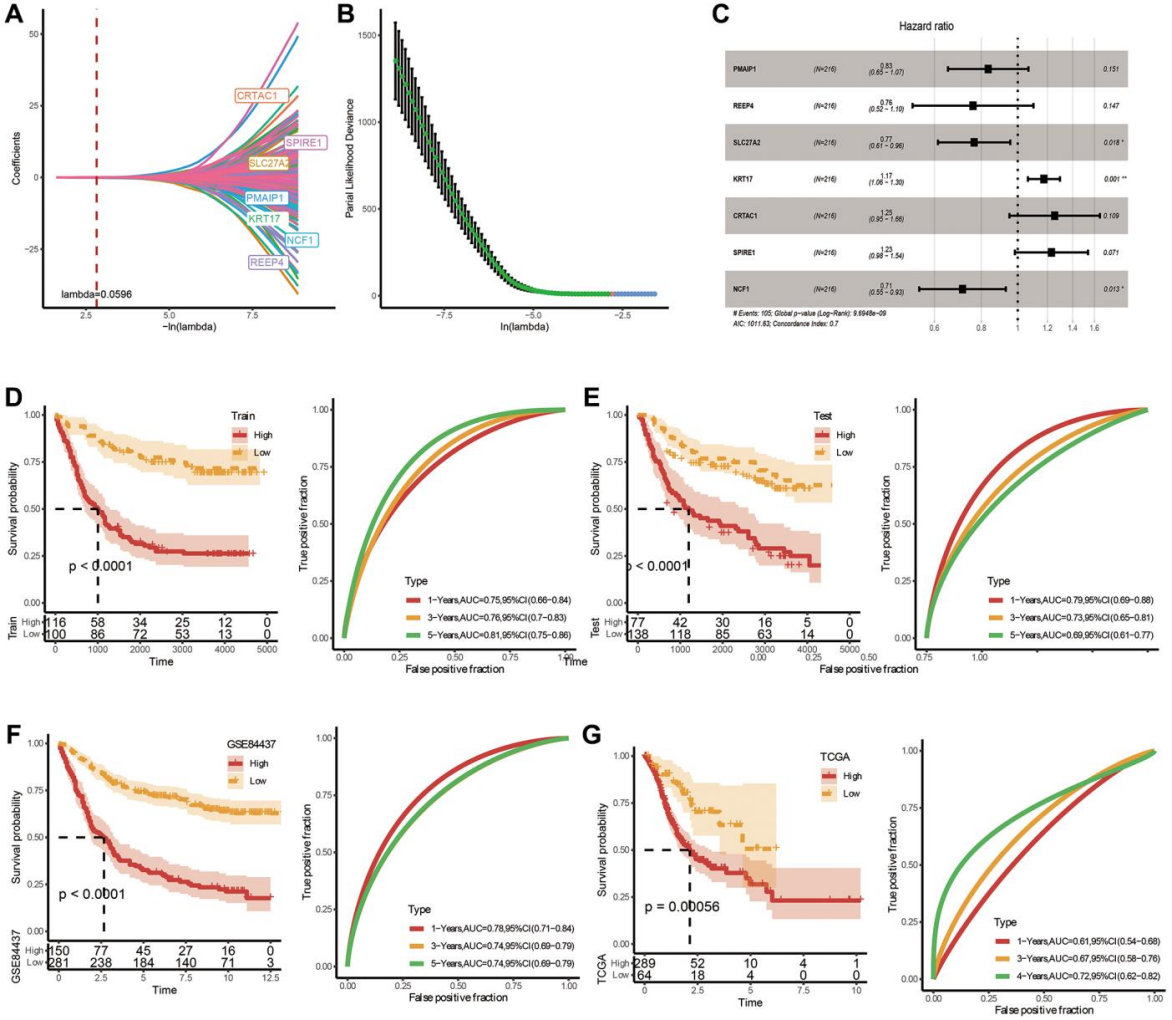
**Construction and validation of a prognostic risk signature in TCGA-STAD based on hub FAM-related genes.** (**A**) Lambda trajectory of differentially expressed genes. (**B**) Confidence interval under lambda. (**C**) Forest map of FAM-related hub genes. (**D**) ROC and K-M survival analysis of riskscore in GSE84437-train dataset. (**E**) ROC and K-M survival analysis of riskscore in GSE84437-test dataset. (**F**) ROC and K-M survival analysis of riskscore in TCGA dataset. (**G**) ROC and K-M survival analysis of riskscore in GSE84437 dataset.

To accurately evaluate the risk of each STAD patient, a risk prognosis model was set up using the intersection of 1721 differentially expressed genes between C1 and C2 using the TCGA and GSE84437 datasets. We randomly divided GSE84437 dataset into train and test datasets (1:1 ratio). Via univariate COX analysis and Lasso method, seven vital genes were identified, and the hazard ratio of these genes was shown in Figure 4A–4C. Next, we built a 7-gene riskscore signature and stratified patients into high and low-risk groups on the base of median riskscore. riskscore = −0.181 × PMAIP1 −0.274 × REEP4 −0.267 × SLC27A2 + 0.161 × KRT17 + 0.227 × CRTAC1 + 0.207 × SPIRE1 −0.338 × NCF. In the meantime, we confirmed the model in three validating groups (the testing set, the entire GSE84437 and TCGA datasets), respectively, and similar results were obtained (Figure 4D–4G). Results of K-M analysis characterized a higher OS in low-risk group. The high AUC values demonstrated the accuracy of the model in predicting the 1-, 3- and 5-year survival rates in the above datasets, due to higher AUC values (>0.6). The AUC values of 1 year, 3 years and 5 years in the training set are 0.75, 0.76 and 0.81 respectively. The AUC values for 1 year, 3 years and 5 years in the validation set are 0.79, 0.73 and 0.69 respectively. The AUC values at 1, 3, and 5 years in the GSE84437 cohort were 0.78, 0.74, and 0.74, respectively. The AUC values at 1, 3, and 5 years in the TCGA cohort were 0.61, 0.67, and 0.72, respectively. To sum up, the data proved that built riskscore signature had a big latent capacity in predicting the SO of STAD crowds.

### Mutation and clinical characteristics in two risk groups

**Figure 5:**
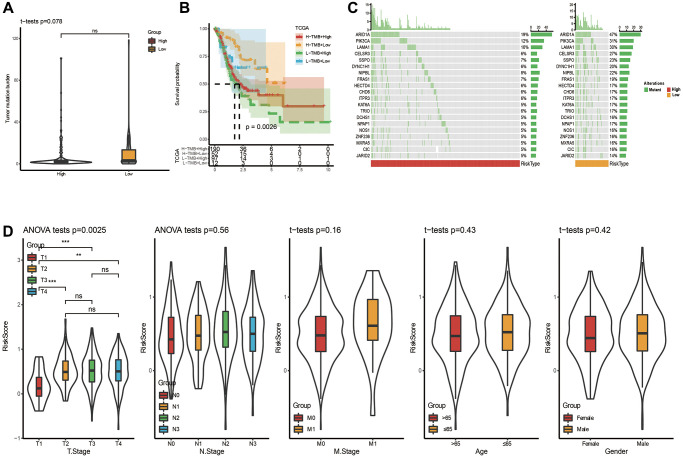
**Somatic mutation analysis and clinicopathological characteristics based on riskscore in TCGA cohort.** (**A**) Analysis of somatic mutation in risk groups. (**B**) Distribution of TMB in risk groups. (**C**) K-M curve combining risk grouping with TMB. (**D**) The distribution of riskscore among different clinicopathological characteristics in the TCGA queue.

The distribution of TMB between two risk groups was carried out, and no obvious difference was seen in Figure 5A. Nevertheless, a significant difference was observed when we combined risk groups with TMB for K-M analysis (Figure 5B). Top 20 gene mutation characteristics in two risk groups from TCGA databases were displayed in Figure 5C. T stage (T1-T4), N stage (N0–N3), M stage (M0-M1), Age (≤65 years or >65 years) and Gender (female or male) are five basic clinicopathological characteristics, which were used to describe differences in riskscores among clinical subgroups. Applying the TCGA dataset, we subsequently investigated the association between clinical characteristics and the riskscore. The riskscore was obviously different among tumor and T stages, and the riskscore was higher in more advanced STAD (Figure 5D), although the differences in riskscore by, N stage, M stage, Age and Gender were not significant (Figure 5D). These analyses indicated that the FAMRGs riskscore signature had clinical significance.

### Construction and estimation of the nomogram

**Figure 6:**
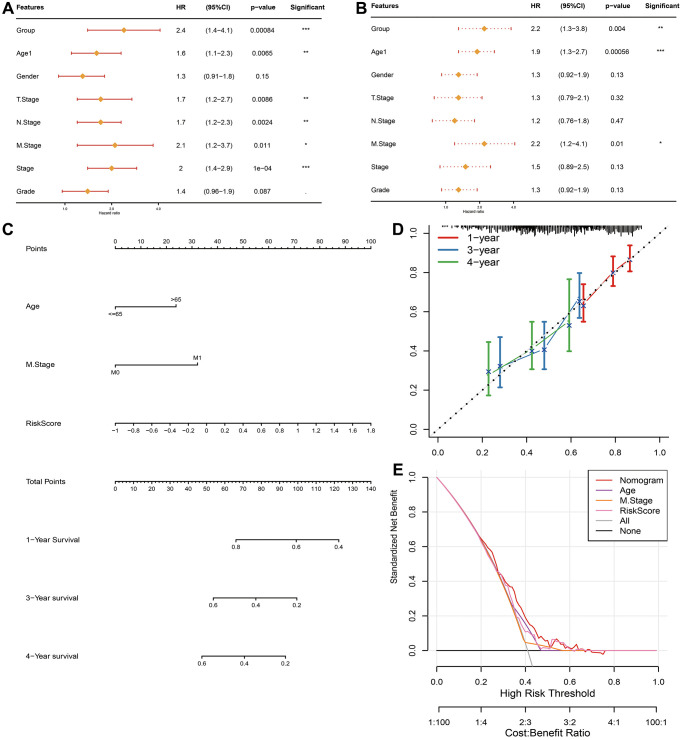
**Establishment and assessment of the nomogram in TCGA-STAD queue.** Forest plot of the (**A**) univariate and (**B**) multivariate Cox regression analyses. (**C**) The nomogram plot was constructed based on Age, M stage and FAMRGs riskscore. (**D**) Calibration plot of the nomogram. (**E**) DCA of the nomogram for 1-, 2- and 4-year OS.

In addition to FAMRGs riskscore model, Age, Gender and TNM stage are also prognostic indicators for STAD. It was expected to check if FAMRGs riskscore could be treated as an independent prognostic indicator. In TCGA-STAD queue, univariate Cox analysis represented that established FAMRGs riskscore, Age, T stage, N stage, M stage and Stage were all markedly associated with prognosis (Figure 6A). Multivariate Cox regression analysis implied that FAMRGs riskscore signature, Age, and M stag were noticeably correlated with prognosis (Figure 6B). The analysis suggested that FAMRGs riskscore model was an independent prognostic indicator for STAD patients.

We constructed a nomogram using TCGA-STAD cohort, by integrating FAMRGs riskscore model, Age and M stage indicators (Figure 6C). The calibration curves illuminated a favorable degree of compliance between the predicted and actual survival time at 1-, 3- and 4-year OS rates (Figure 6D). Furthermore, the DCAs illustrated that the nomogram did well in forecasting prognostic benefits of STAD patients (Figure 6E).

### Pathway scoring in risk grouping related to the progression of tumor

**Figure 7:**
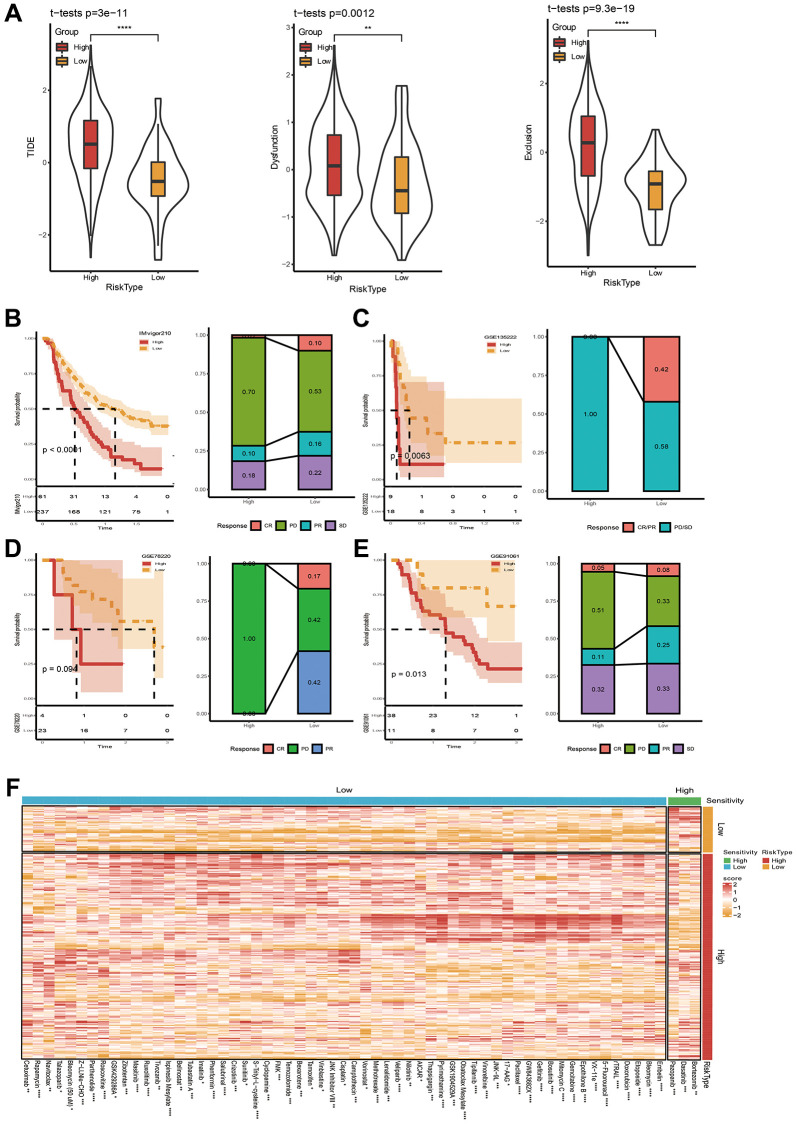
**Immunotherapy and drug sensitivity assessment.** (**A**) TIDE score between high- and low-risk groups. (**B**–**E**) Riskscore survival curve and immunotherapy distribution in IMvigor210, GSE135222, GSE78220 and GSE91061 datasets. (**F**) Differential heatmap analysis of 62 chemotherapeutics in high and low riskscore groups. ^*^p < 0.05, ^**^p < 0.01, ^***^p < 0.001, ^***^p < 0.0001.

We downloaded 41 pathways in ‘h. all. v7.4. symbols. Gmt’ file from the GSEA website, scored them using ssGSEA method, and compared them in two risk groups of TCGA-STAD cohort. 39 pathways showed marked differences as presented in Figure 7A. Then the relevance between these 41 pathways and our riskscore was calculated. The riskscore was positively correlated with TGF_ BETA_ SIGNALING, MYOGENESIS, APICAL_JUNCTION and EPITHELIAL_MESENCHYMAL_ TRANSITION pathway with a *P*-value < 0.05 (Figure 7B).

In another way, we obtained 13 pathway markers or pathways related to tumor from a reference [40]. Then, we calculated the Pearson relevance between riskscore and ssGSEA score of these gene markers or pathways. We found that riskscore positively correlated with Homologous recombination, DNA replication, and Base error repair (Figure 7C). Besides, EMT1, EMT2, EMT3, WNT target, FGFR3 related, Cell cycle had higher scores in high riskscore group (Figure 7D).

### Immunotherapy and drug sensitivity analysis applying FAMRGs riskscore

After confirming the performance of the 7-gene signature in describing clinical characteristics patients with STAD. Studies upon immunotherapy were also conducted. Here, we firstly applied TIDE software to estimate the potential for immunotherapy in two risk groups. In Figure 7A, it can be seen that the TIDE score was lower in low-risk group, indicating a higher likelihood of immunotherapy and more possibility to benefit from immunotherapy. Moreover, the Dysfunction score and Exclusion score are still lower in low-risk group (Figure 7A). Correlations between riskscore and TIDE, Dysfunction, and Exclusion were simultaneously executed, and the results unveiled that our riskscore showed a significant positive correlation with TIDE, Exclusion, and Dysfunction (Supplementary Figure 2). Immunotherapy treated data (IMvigor210, GSE135222, GSE78220, and GSE91061) were also applied. We employed our method to calculate riskscore scores, and obtained the optimal cutoff K-M curve. The results showed the proportion of progressive disease (PD)/stable disease (SD) in the high-risk group was higher (Figure 7B–7E). All above data collectively indicated that the possibility of high-risk grouping gaining benefits from immunotherapy are relatively low.

At the same time, we studied the sensitivity of 61 traditional chemotherapy drugs in two risk groups. The results exhibited that 3 drugs were sensitive to high-risk groups, while the rest 59 drugs were sensitive to low-risk groups (Figure 7F). Taken together, the study might demonstrate why patients with low riskscore have a good prognosis and why patients with low risk often exhibit a better response to immunotherapy.

### The performance of riskscore model in pan cancer

**Figure 8:**
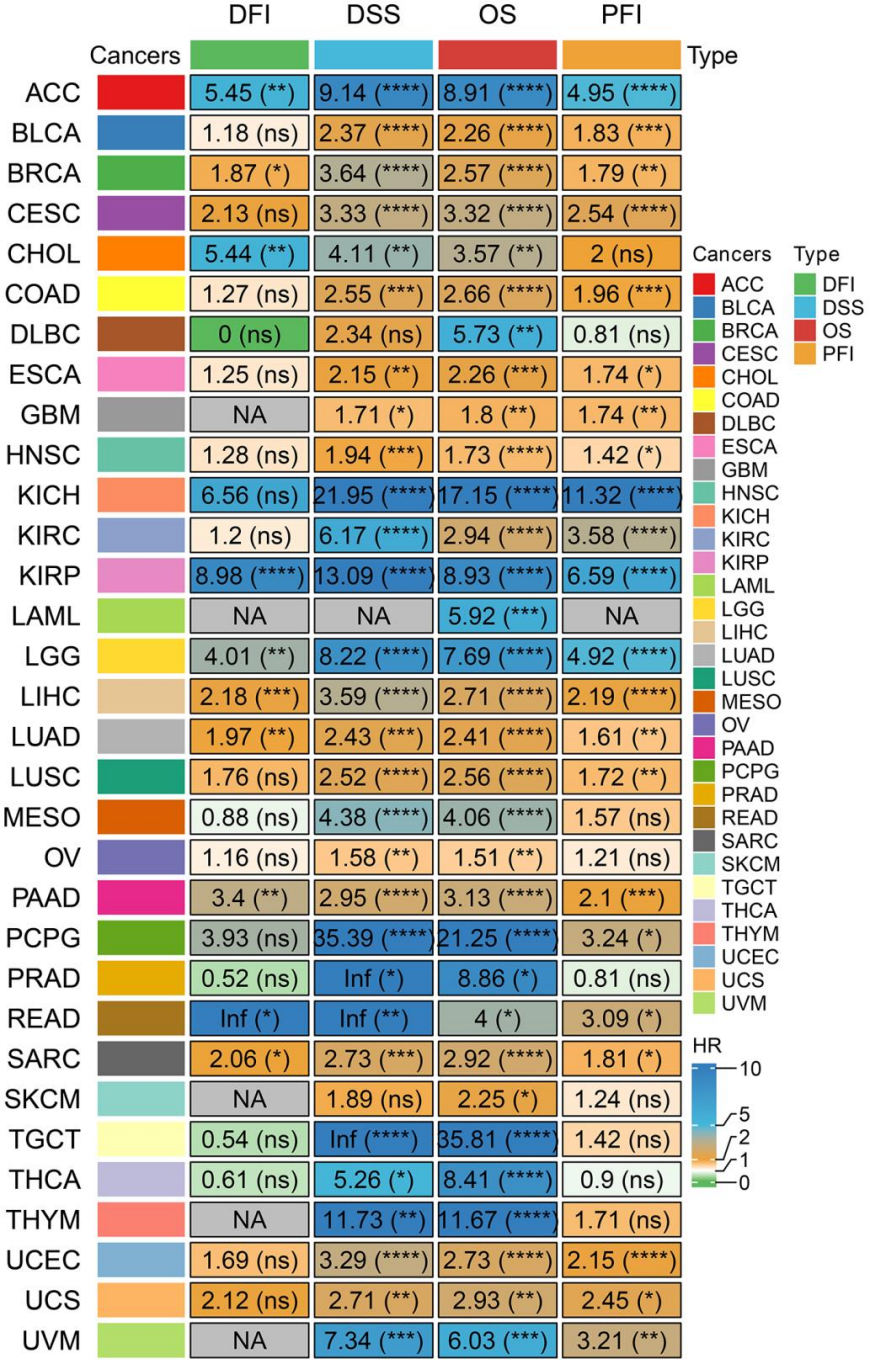
**Prognostic analysis of our riskscore signature at different times in pan cancer.** (The numbers inside represent the HR value, and the ^*^in parentheses after it represents the log Rank *P*-value of HR. ^*^P < 0.05, ^**^P < 0.01, ^***^P < 0.001, and ^****^P < 0.0001. The gray NA represents that there is no corresponding survival time and status in the tumor or that HR values cannot be calculated).

We further expanded cancer range, and applied the riskscore model to calculate the riskscore of the remaining 32 types of cancers in TCGA database, and carried out K-M analysis based on LogRank method under four time and state of OS, Progression free survival (PFI), Disease free interval (DFI), and Disease specific survival (DSS). As seen in Figure 8, under OS time and status, our riskscore model showed intense discrepancy between high and low riskscores among 32 cancer species. Furthermore, riskscore showed notable differences at least 10 cancer species (under DFI time and state). This result indicates that our riskscore also has the potential to be used for prognostic evaluation in other cancer models. These studies suggest that the riskscore model we established may be useful for future cancer treatment and prognosis research.

## DISCUSSION

STAD is a fatal cancer with unfavorable therapeutic response and survival rate. Factors (as such low diagnostic rates at an early stage, high degree of intratumor heterogeneity, together with drug resistance) result in the unfavorable survival outcomes of STAD patients [41–43]. A growing number study has suggested that the FAM pathway showed importance in tumor progression and possesses pro-tumor potentials [10, 44]. Exploring FAM-relevant subtyping in STAD cohorts will help disclose distinct states of the tumor and implement precise therapy strategies.

In present research, we firstly demonstrated dysregulated FAM in STAD. Subsequently, 14 FAM-related coding genes were significantly correlated with prognosis. Then they were applied for molecular typing. Two subtypes defined as C1 and C2 clusters displayed differentiated prognosis. Immune infiltration and GSEA analysis disclosed that C2 subtype with worse OS had a higher immune score, and was tightly correlated with HYPERTROPHIC_CARDIOMYOPATHY_HCM pathway and DILATED_CARDIOMYOPATHY pathways, which were also reported in another STAD article [45] based on fatty acid-related molecular typing. The CIBERSORT analysis revealed that in C2 subtype, the levels of immature and activated B cells, and activated dendritic cells, central memory CD4 and CD8 T cells, microphages, natural killer T cells as well as mast cells were significantly higher. These results were similar to others’ findings [46, 47]. These immune results indicated that cluster C2 owns an intense immunosuppressive tumor microenvironment (TME). In terms of cancer immunoediting theory, tumor cells in immune escape state can circumvent the damage of immune cells and are highly malignant, which ultimately leads to lower patient survival rates [48]. In advanced stage, most tumor cells can secrete TGF-β. Elevated TGF-β can prevent immature T cells from differentiating into Th1 cells, and promotes T cells to transform into Treg subpopulations, and inhibits the antigen presentation function of dendritic cells, finally leading to immune escape of tumor cells [49]. Therefore, in the riskscore signature established on the basis of subtypes, riskscore was positively correlated with TGF_BETA_SIGNALING pathway, may also indirectly explain the immune escape of tumor cells.

As one of the most essential characteristics in malignant tumors, distant metastasis is a comprehensive process, which is responsible for over 90% mortality of cancer-related [50, 51]. During this process, tumor epithelial-mesenchymal transition (EMT) has a substantial impact on tumor cell metastasis and diffusion at an early stage, that is proved to be the main process of human gastric cancer [52]. In our study, a FAMRGs riskscore signature was established and evaluated its biologic function. We disclosed that in high riskscore group, EMT1, EMT2, EMT3, WNT target and FGFR3 related pathway score were higher. It’s well-known that these marker genes or pathways participated in regulating the EMT process. On the other hand, the riskscore was obviously higher in more advanced STAD patients. These two findings imply that FAMRGs riskscore signature could help identify the disease development status of STAD patients.

Seven FAMRGs (PMAIP1, REEP4, SLC27A2, KRT17, CRTAC1, SPIRE1, NCF1) were utilized to construct a riskscore model. The riskscore signature was demonstrated to be an independent factor for prognosis of STAD patients, and was able to differentiate the sensitivity of patients to prevalent therapeutic drugs. PMAIP1 is a P53 response gene and acts as a protective factor because its facilities activation of the apoptotic program in mitochondria, and the suppression of PMAIP1 could lead to venetoclax resistance in acute myeloid leukemia cell line [53, 54]. In the meantime, the downregulation of PMAIP1 resulted in cell multiplication and cell motility in non-small cell lung cancer [55]. REEP4 is a known endoplasmic reticulum membrane protein, and was identified as a key morphogenetic factor in coordinating nuclear envelope reformation with mitotic nuclear pore complexes biogenesis [56, 57]. Interestingly, it was the first time, that REEP4 was discovered in STAD disease. SLC27A2 is a fatty acid transporter [58]. Recent research uncovered that SLC27A2 may suppress the epithelial-to-mesenchymal transition through SLC27A2-CDK3-EMT axis [59]. While, in another research, SLC27A2 showed cancer promoting properties. Tao et al. [60] found that suppression of SLC27A2 could weaken neuroblastoma survival, as such inhibiting tumor growth, extending survival time, and exerting synergistic anti-tumor effects in animal experiments. The complex function of SLC27A2 needs deepening studies. KRT17 is a type I intermediate filament. The initial multiple data research showed that KRT17 may be regarded as one of the prognostic biomarkers for gastric cancer [61]. Newly published research focused on diffuse gastric cancer, demonstrated that KRT17/YAP/IL6 axis contributed to maintaining E-cadherin loss, EMT feature, and metastasis of this cancer [62]. These two papers suggest an important role of KRT17 in STAD. Through bioinformatics data analysis, CRTAC1 was also identified as 1 of 8 key genes for a risk model used for gastric adenocarcinoma prognosis [63], strengthening the significance of this gene in STAD research. Neutrophil cytosolic factor 1 (NCF1), is a subunit of the NADPH oxidase 2 complex. The researches about NCF1 variant were mainly related to autoimmune diseases such as lupus erythematosus [64, 65]. Nowadays, utilizing bioinformatics analysis tools, From the brown module, which indicated a strong connection to genetic connectivity, NCF1 was found as a pivotal gene, which can be treated as a bio-markers for diagnosing early lung cancer [66]. Surprisingly, NCF1 was also screened as a hub gene in a necroptosis riskscore signature for gastric cancer [67]. However, the role of SPIRE1 in cancer is a paucity of studies.

Based on the currently known functions of these seven genes. As a P53-responsive gene, PMAIP1 plays a key role in the apoptosis process. In STAD, PMAIP1 may play a protective role in inhibiting tumor growth by enhancing the apoptotic response. However, PMAIP1 may be inactivated in some gastric adenocarcinoma cells due to abnormalities in the P53 pathway, leading to increased resistance to chemotherapy drugs. Therefore, we speculate that PMAIP1 may be an important marker for predicting STAD treatment response and developing new treatment strategies. The discovery of REEP4 provides a new perspective that endoplasmic reticulum membrane proteins may play a role in cell division and nuclear structure reorganization in STAD. Abnormal expression of REEP4 may lead to abnormal cell cycle regulation, thereby promoting disordered proliferation and metastasis of STAD cells. SLC27A2 As a fatty acid transporter, SLC27A2 plays a dual role in regulating lipid metabolism in tumor cells. In STAD, SLC27A2 may promote or inhibit tumor progression by affecting lipid metabolism. This bidirectional effect may depend on specific cellular context and microenvironmental conditions, indicating that the role of SLC27A2 in STAD is variable and complex. KRT17 plays a key role in maintaining cell structure and signaling. In STAD, KRT17 may promote inflammatory response and EMT characteristics by activating the YAP/IL6 axis, thereby enhancing tumor invasiveness and metastasis. Therefore, KRT17 may be a driver of STAD progression and metastasis. The role of CRTAC1 in the prognosis of gastric adenocarcinoma suggests that it may affect the tumor microenvironment or interact with specific cell signaling pathways. The expression of CRTAC1 may be related to cell adhesion, migration or immune evasion mechanism in STAD, affecting tumor growth and immune response. As part of the NADPH oxidase complex, NCF1 plays an important role in generating reactive oxygen species (ROS) and regulating immune responses. In STAD, abnormal expression of NCF1 may lead to oxidative stress response and changes in the immune microenvironment, promoting tumor cell survival and immune evasion. Although there are fewer studies on SPIRE1 in cancer, its role in cytoskeletal reorganization and organelle trafficking suggests that it may play a role in the migration and invasion of STAD cells. Changes in the expression of SPIRE1 may affect the dynamic behavior of tumor cells and thus play a role in the metastasis process of STAD. These speculations provide a theoretical basis for future experimental verification and may reveal new therapeutic targets and strategies to improve the prognosis and therapeutic effects of STAD patients.

Immune checkpoint inhibitors (ICIs) bring bright to patients with advanced cancer because of their powerful effect and less side effects. Only a few patients benefit from this method, yet. As a result, there is imperative demand to select the crowd with a high response rate for precise therapy.

Our study revealed that the low-risk group had a tendency towards higher TMB (*p* = 0.078). In line with previous findings, there is an independent predictive relationship between high TMB and good response to immune checkpoint inhibitor therapy in different types of cancer. There is an independent predictive relationship between high TMB and favorable response to immune checkpoint inhibitor therapy in different types of cancer [68]. High TMB was shown to be a predictor of treatment efficacy with nivolumab plus ipilimumab, suggesting that patients with high TMB may benefit from this combination immunotherapy [69]. Another study also found that patients with higher TMB were more likely to benefit from PD-L1 inhibitor treatment [70]. This is consistent with the trend of our study results. Patients in the low-risk group had higher TMB and a higher degree of response to immunotherapy. Except for TMB, TIDE score is an innovative immunotherapy biomarker to ICIs [35]. The higher TIDE score, the bigger chance to tumor immune escape [71]. In other words, the lower the TIDE score, the better therapeutic potential to ICIs. Current research showed that STAD patients in low-risk group had lower TIDE scores, were more likely to receive immunotherapy and benefit more from it.

One limitation for this research is that it’s a bioinformatic analysis based on two public datasets. The practical prognostic values of the FAMRGs riskscore model should be confirmed in independent and multicenter STAD cohorts with larger sample sizes in the future.

## CONCLUSION

Taken together, we applied FAM-derived genes to establish a FAMRGs riskscore signature, which displayed powerful advantages in the prognosis evaluation of STAD patients. FAMRGs riskscore signature constructed in the current study could offer a valuable viewpoint and a helpful way for deeply exploring the role of FAM and targeting immunotherapy during cancer progression in STAD.

## Supplementary Materials

Supplementary Figures
