# Supplementary material for: Identification of a fatty acid metabolism-related gene signature to predict prognosis in stomach adenocarcinoma
Source: Aging (Albany NY). 2024 May 13;16(10):8552–71. doi: 10.18632/aging.205823 (PMC11164501; doi:10.18632/aging.205823)
Supplement: Supplementary Figures [file aging-16-205823-s001.pdf]

SUPPLEMENTARY FIGURES

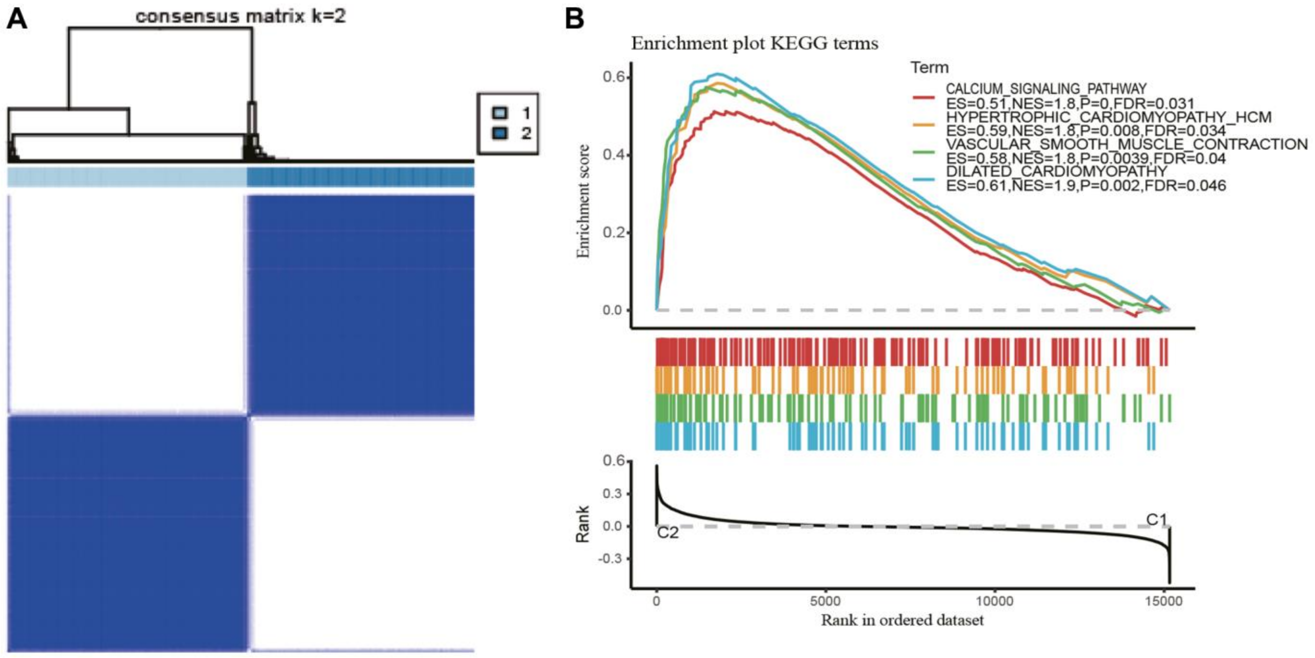

Supplementary Figure 1. Identification of molecular subtypes and gene set enrichment analysis. (**A**) Heatmap of sample clustering when k = 2 in GSE84437 dataset. (**B**) GSEA pathways score analysis between C1 and C2 in GSE84437 dataset.

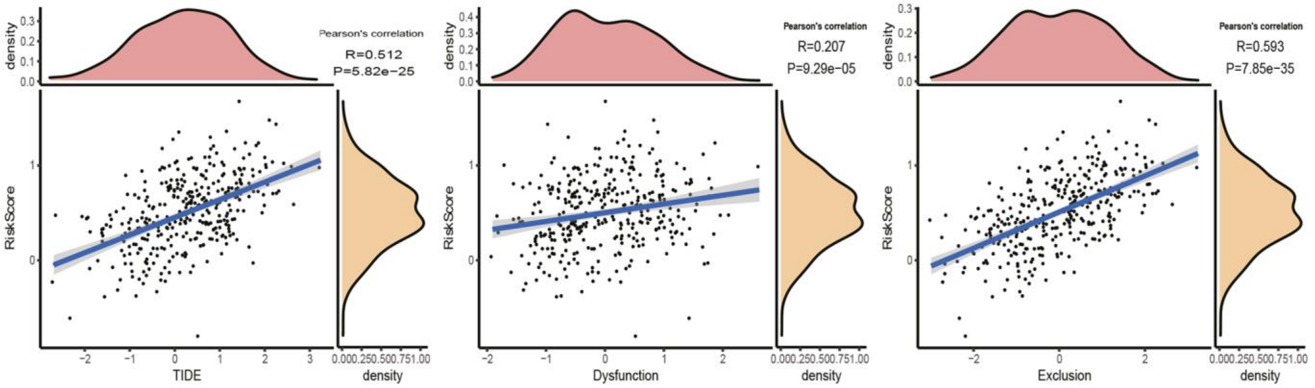

Supplementary Figure 2. Scatter plots of correlation analysis between TIDE score and riskscore.
